# Exploring opportunities to integrate climate change into Gross National Happiness for Bhutan and its application for global wellbeing-centred Climate Resilient Development

**DOI:** 10.1007/s00267-026-02501-5

**Published:** 2026-05-28

**Authors:** Tashi Dorji, Angus Morrison-Saunders, David Blake

**Affiliations:** 1 School of Science, Edith Cowan University, Perth, WA Australia; 2 Centre for People Place and Planet, Edith Cowan University, Perth, WA Australia; 3 Research Unit for Environmental Sciences and Management, North-West University, Potchefstroom, South Africa

**Keywords:** Bhutan, Climate change, Climate resilient development, Gross national happiness, Holistic development

## Abstract

Climate change poses an increasing threat to human wellbeing, but despite this intricate relationship, addressing climate change rarely mainstreams wellbeing objectives. This study explores opportunities to integrate climate change into Bhutan’s development philosophy of Gross National Happiness (GNH) and examines how this experience can inform a wellbeing-centred global application through Climate Resilient Development (CRD). Using a qualitative design combining semi-structured interviews with 41 policy influencers in Bhutan, document analysis, and literature synthesis, this study identifies two complementary points of integration. The first involves identifying and then embedding climate-wellbeing stressors into the 9 domains and 33 GNH indicators used in the nationwide survey that constructs the GNH index. The second focuses on identifying and integrating climate-wellbeing stressors into the GNH screening tool through the 23 determinants used in assessing policies. Building on Bhutan’s GNH and climate experience, this study identifies six global pathways to operationalise wellbeing-centred CRD through strengthening governance and leadership, embedding wellbeing metrics into climate policy instruments, advancing knowledge pluralism and participatory co-production, linking local resilience to global frameworks, mobilising finance for wellbeing-oriented climate action, and multi-level integration. This study positions GNH as a globally relevant guide for transforming climate action towards human and planetary flourishing and offers an integrative approach for nations pursuing wellbeing objectives to combine these with climate action for holistic development. Its global relevance lies in offering a wellbeing-centred approach to CRD.

## Introduction

The intensifying impacts of climate change are reshaping the conditions for human wellbeing the world over (Lamb & Steinberger, 2017). Climate impacts like rising temperatures, erratic rainfall, biodiversity loss and the escalation of floods, droughts, and storms are causing disruptions beyond the material, and it is changing the way people experience the sense of security, belonging, and wellbeing (Adger et al., 2022). Taking note of the multidimensional impacts of climate change, the Intergovernmental Panel on Climate Change (IPCC, 2023) calls for a more holistic approach to addressing climate change in its 2022 Sixth Assessment Report (AR6). It advocates nations to embed equity, social justice, and human wellbeing into climate policy through Climate Resilient Development (CRD), which is a multidimensional process combining mitigation, adaptation, and sustainable development in an integrated manner to reduce systemic vulnerabilities while enhancing human and ecological wellbeing (Schipper et al., 2022). Nonetheless, translating wellbeing concepts into governance and policy practice remains a global challenge (Schipper et al., 2022).

While a growing body of research recognises the links between climate change and human wellbeing, the literature remains fragmented across disciplines. Existing studies often examine specific dimensions—such as health impacts, livelihood disruptions, or psychological stress—in isolation rather than within an integrated framework of wellbeing (Adger et al., 2022; Doherty & Clayton, 2011). Similarly, adaptation and resilience scholarship emphasises the importance of social equity, local context, and diverse knowledge systems, including Indigenous and community-based approaches (Ensor et al., 2016; Schipper et al., 2022). However, these strands of research rarely converge into coherent models that embed wellbeing within climate governance systems. Consequently, there is limited practical guidance on how to operationalise climate–wellbeing linkages within national development frameworks and policy processes (O’Brien & Selboe, 2015). This gap highlights the need for integrative approaches that move beyond conceptual recognition towards actionable governance mechanisms that align wellbeing objectives with climate action.

An initial phase of the broader research, of which this paper forms a final component, comprised two systematic literature reviews of global evidence on climate change and wellbeing (Dorji et al., 2023, 2024), alongside two empirical studies examining Bhutan’s experience (Dorji et al., 2025, 2026). Key findings from the first literature review (Dorji et al., 2023) reveals that climate change impacts on wellbeing are complex and multidimensional and calls for adaptation and resilience strategies that are socially just, locally grounded, context-specific, and holistic. Further, where Indigenous Peoples are involved, key additional climate change considerations for wellbeing and adaptation are that Indigenous Knowledge (IK) offers local and time-tested adaptive approaches for managing land, water, agriculture, and biodiversity to deal with climate impacts with substantial implications for wellbeing (Dorji et al., 2024). Integrating IK with climate change and wellbeing also warrants embracing more holistic approaches to development. Together, the two papers (Dorji et al., 2023, 2024) show that wellbeing and resilience are mutually reinforcing—wellbeing of communities is more sustainable when it has the capacity to adapt, and communities adapt more effectively when wellbeing exists (Becvarik et al., 2025). This reciprocal relationship shows that wellbeing should be a core component in designing and assessing climate action contrary to the conventional separation of climate action and wellbeing studies (Adger et al., 2022). The insights point to the need to integrate climate change and wellbeing objectives by adopting strategies that are context-specific and socially equitable (Schipper et al., 2022).

The empirical studies from Bhutan drew on insights from 41 policy influencers consisting of political leaders, civil servants, NGO representatives and media editors (further details in methodology section later). This included an evaluation of gross national happiness (GNH) as a development model and a governance tool in Bhutan (Dorji et al., 2025) and Bhutan’s experience with addressing climate change (Dorji et al., 2026). Key findings reveal both the promise and fragility of GNH in addressing climate change. While GNH provides a multidimensional and comprehensive governance framework embedding beyond-GDP dimensions like psychological wellbeing, time use, culture, and community vitality (Ura, 2022; Ura et al., 2012 Thinley and Hartz-Karp, 2019), its translation into practice is constrained by the absence of binding policy mechanisms, limited resources, and the inconsistent application of GNH. Moreover, it exposes Bhutan’s climate change paradox as the world’s only carbon negative country (Bhattarai, 2021) yet being highly vulnerable to climate impacts because of its location in the fragile Himalayan region (Hoy et al., 2015; Chhogyel et al., 2020) highlighting tensions between aspiration and exposure. The findings, therefore, points out that for GNH to realise its transformative potential to reframe climate change as a wellbeing issue, it must work towards a coherent integration of climate change and GNH.

GNH represents Bhutan’s distinctive approach to development, positioning wellbeing as the central objective of governance (Ura, 2022; Ura et al., 2012,). Emerging in the 1970s and later embedded in Bhutan’s 2008 Constitution, GNH reflects a values-based philosophy grounded in holistic and collective notions of wellbeing (Thinley and Hartz-Karp, 2019; Ura, 2022). It is operationalised through a multidimensional framework comprising four pillars and measured using the GNH Index, which includes nine domains and 33 indicators capturing diverse aspects of human and societal wellbeing (Ugyel et al., 2023; Ura, 2022). Beyond measurement, GNH also functions as a governance tool, informing national planning processes and policy appraisal mechanisms. However, scholarship highlights persistent challenges in its practical implementation, including inconsistent application, limited institutionalisation, and tensions between wellbeing objectives and economic development priorities (Dorji et al., 2025). These characteristics underscore both the relevance of GNH and the need to strengthen its integration with climate change and wellbeing governance.

This paper extends the foundations laid by the broader research on the relationship between climate change and wellbeing by examining how climate change can be coherently integrated with GNH. It addresses two research questions: (i) What are the opportunities to integrate climate change into the GNH framework? and, (ii) How to use the insights from Bhutan’s experience for global application of wellbeing-centred climate resilient development? The next section explains the research methodology, outlining the qualitative approach used to examine integration opportunities. It is followed by an integrated results and discussion section presenting findings for the two research questions, focusing on integrating climate change into GNH and its implications for global wellbeing-centred Climate Resilient Development (CRD). The conclusion summarises the key contributions and outlines implications for policy and future research.

## Methodology

This paper employed a qualitative research design combining semi-structured interviews, document analysis and literature synthesis. Guided by the two research questions outlined previously, this section outlines the methods in two complementary components.

### Methods in integrating climate change into the GNH framework

This component drew primarily on empirical evidence from Bhutan and document analysis. Semi-structured interviews were conducted in Bhutan over two months in early 2024 with 41 policy influencers, including politicians, civil servants, NGO leaders, and media professionals. A detailed breakdown of participants by professional background and expertise is provided in Table 1. Participants were selected using a purposive sampling approach, targeting individuals with direct experience and expertise in GNH and climate change, based on their roles in policy, governance, and public discourse. Data saturation was reached when no new themes or insights were emerging from additional interviews. The first author conducted all the interviews between February and April of 2024. The interviews pursued three lines of inquiry: (i) the efficacy of GNH application (reported in Dorji et al., 2025); (ii) Bhutan’s experience with addressing climate change (reported in Dorji et al., 2026); and (iii) opportunities for integrating climate change and GNH—which is the focus of this paper. These three lines of inquiry were designed to generate both contextual understanding and practical insights on the intersection of climate change and GNH.

**Table 1:** The professional background and related expertise in climate change and GNH of the 41 participants

Occupation/Profession		Participant numbers	Total
1.	Civil Servants (S)	1, 2, 3, 8, 10, 12, 13, 15, 17, 20, 21, 23, 25, 29, 30, 31, 33, 34, 36, 39, 40, 41	22
2.	Politician (P)	2, 6, 9, 11, 13, 17, 22, 28, 29, 30, 31, 32, 40, 41	14
3.	Non-government organisation (N)	6, 9, 10, 11, 12, 16, 18, 19, 20, 22, 24, 25, 26, 27, 31, 35, 37, 38, 39	19
4.	Media (M)	2, 3, 4, 5, 7, 10, 14, 16, 19, 21, 28, 30, 32	13
Expertise			
1.	GNH (G)	8, 20, 21, 29, 30, 35, 41	7
2.	Climate change	1, 12, 15,18, 23, 24, 25, 26, 31, 33, 36, 39, 41	13

For this paper, the interview data were analysed thematically in NVivo 12 (Phillips & Lu, 2018) to identify opportunities for integrating climate change into the GNH framework and informing its global application through CRD. The analysis followed an inductive coding process, where relevant responses were first coded and then grouped into categories based on similarities. These categories were further refined into sub-themes and overarching themes that captured key integration entry points. The themes derived from the interviews were used to establish the rationale for integration and identify key entry points—particularly the GNH Index and the GNH Policy Screening Tool—which guided the subsequent document analysis. The data were also re-analysed to identify broader governance and implementation themes, which were compared with CRD principles from the IPCC literature, leading to the development of six pathways for operationalising wellbeing-centred CRD.

Towards integrating climate change into the GNH framework, a qualitative document analysis (Bowen, 2009) of peer-reviewed literature was conducted to identify the relationship between each of the 33 indicators and climate change. To accomplish this, the first step involved searching articles indexed in Scopus and Google Scholar using combinations of the keywords “climate change” OR “global warming” AND “Bhutan” AND each indicator term. Scopus was selected to ensure the retrieval of strictly peer-reviewed literature, while Google Scholar was additionally used to capture both peer-reviewed and relevant grey literature, thereby broadening the evidence base. For each indicator term, the first 10 most relevant results were reviewed to establish the climate–wellbeing relationship. Studies were included if they were peer-reviewed or high-quality grey literature, written in English, and demonstrated explicit or inferable linkages between climate change and the relevant GNH indicators or determinants, while duplicates, non-English sources, low-rigour materials, and irrelevant studies were excluded.

**Fig. 1:**
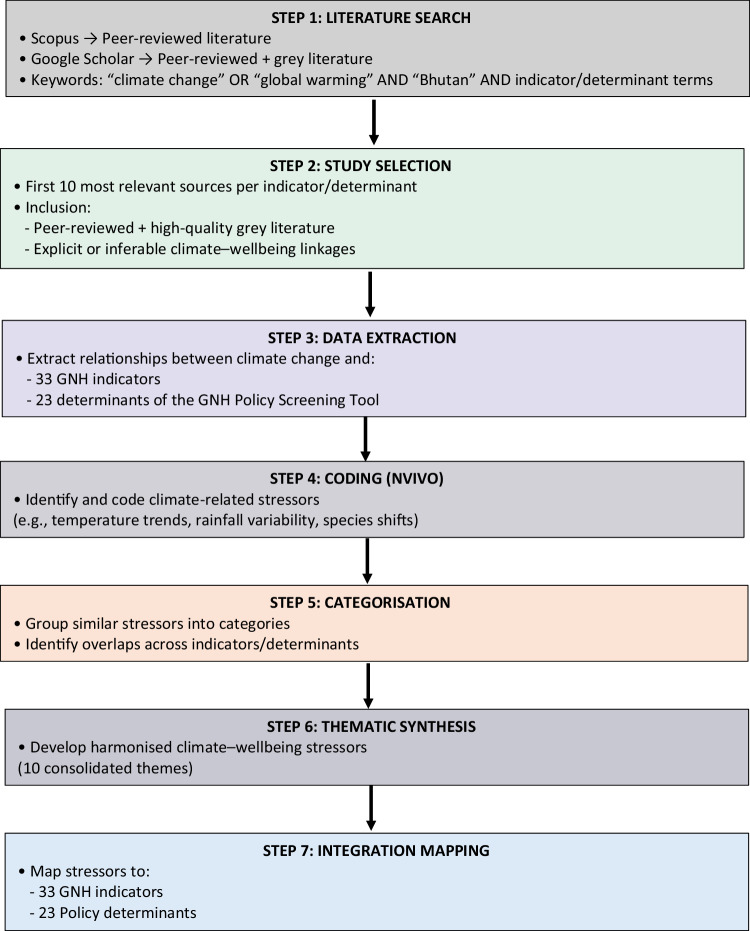
Stepwise process of qualitative document analysis for integrating climate change into Bhutan’s GNH framework. The figure illustrates the sequential process from literature search and study selection to data extraction, coding, categorisation, thematic synthesis, and integration mapping. The process results in the development of harmonised climate–wellbeing stressors, which are embedded into GNH indicators (measurement) and policy screening determinants (evaluation).

The analysis followed a clearly defined stepwise thematic procedure. First, relevant studies were reviewed to extract evidence on how climate change influenced each GNH indicator, and these relationships were recorded (column 4, Table 2). Second, climate-related stressors—such as temperature trends, rainfall variability, and species range shifts—were identified and coded in NVivo (column 5, Table 2). Third, an inductive coding process was applied, whereby similar stressors were grouped into categories based on conceptual similarities. Fourth, these categories were iteratively refined and synthesised into higher-order themes, resulting in harmonised climate–wellbeing stressors (Table 3). This process reduced overlapping stressors into a smaller set of analytically coherent themes. Finally, these harmonised stressors were mapped onto each relevant indicator to complete the integration process. A schematic diagram (Fig. 1) illustrates each step of this analytical process to enhance clarity and replicability.

**Table 2:** Relationship between 9 domains, 33 indicators of GNH and climate change, and harmonised climate-wellbeing stressors that could be integrated into each indicator

	9 Domains	33 Indicators	Relationship with Climate Change	Climate-related stressors defining the relationship between the indicator and climate change	Harmonised climate-wellbeing stressors to be integrated into the indicator
1.	Ecological diversity & resilience	Wildlife change	Rising temperatures and altered monsoon rainfall are shifting vegetation zones and species ranges, leading to increased human–wildlife conflict and biodiversity stress (Penjor et al., 2022; Sharma et al., 2020; Chhogyel et al., 2020)	Temperature trend, rainfall variability, and species-range shift variables	Temperature Stress; Hydro-climatic Variability; Ecosystem & Biodiversity Stress
		Urbanisation issues	Climate-driven crop failures, water scarcity, and land degradation accelerate rural-to-urban migration and expose urban areas to floods and heat stress from extreme rainfall (Jamtsho et al., 2025; Chhetri et al., 2024b)	Precipitation intensity, flood recurrence, and migration pressure variables	Hydro-climatic Variability; Disaster Hazard Exposure; Migration & Displacement Pressure
		Responsibility to environment	Increasing forest-fire frequency, prolonged dry seasons, and water scarcity weaken traditional conservation ethics and limit adaptive stewardship (Chhetri et al., 2024b; Delma et al., 2024)	Forest-fire incidence, drought duration, and water-availability index	Disaster Hazard Exposure; Hydro-climatic Variability; Ecosystem & Biodiversity Stress
		Ecological issues	Glacial retreat, erratic monsoons, and slope instability increase soil erosion and sedimentation, threatening hydropower and agricultural productivity (Lama et al., 2024; Khandu et al., 2022)	Embed glacial-melt rate, landslide frequency, soil-erosion index, and river-sediment load	Glacial & Cryosphere Change; Disaster Hazard Exposure; Ecosystem & Biodiversity Stress
2.	Psychological wellbeing	Life satisfaction	Climate-induced livelihood insecurity from floods, droughts, and crop failure reduces perceived happiness and stability, leading to declining life satisfaction (Kamei et al., 2020; Thinley & Hartz-Karp, 2019).	Livelihood security, disaster-recovery stability, and income-loss frequency	Livelihood & Income Instability; Disaster Hazard Exposure; Psychosocial & Emotional Stress
		Positive emotions	Extreme weather events, displacement, and livelihood loss increase fear, anxiety, and trauma while reducing optimism and emotional stability (Wouters & Dema, 2024; Chhetri, et al., 2024a)	Stress frequency, coping capacity, and post-disaster emotional recovery	Psychosocial & Emotional Stress; Disaster Hazard Exposure
		Negative emotions			
		Spirituality	Glacial retreat, floods, and landslides damage sacred sites and interrupt rituals, weakening spiritual connection to nature and cultural landscapes (Dorji, 2024; Yangzom & Wouters, 2024; Phanchung et al., 2022)	Sacred-site vulnerability, ritual disruption frequency, and landscape loss	Infrastructure Disruption; Ecosystem & Biodiversity Stress; Disaster Hazard Exposure
3.	Health	Self-reported health status	Increasing temperature, heatwaves, and water contamination heighten risks of heat stress, diarrhoeal disease, and vector-borne illnesses, affecting overall perceived health (Chhetri et al., 2024a; Ruby et al., 2025)	Temperature rise, heatwave exposure, and vector-borne disease incidence	Temperature Stress; Health & Disease Risk
		Healthy days	Extreme weather, pollution, and food insecurity reduce the number of days individuals feel physically and mentally healthy (Chhetri, et al., 2024a; Ruby et al., 2025).	Air-quality index, extreme-temperature frequency, and food-security stability	Health & Disease Risk; Temperature Stress; Livelihood & Income Instability
		Disability	Floods, landslides, and related disasters cause injuries and long-term disabilities, particularly in rural communities with limited health infrastructure (Dhimal et al., 2021; Chhetri, et al., 2024a)	Disaster-related injury frequency, rehabilitation access, and infrastructure damage	Disaster Hazard Exposure; Infrastructure Disruption
		Mental health	Displacement, livelihood loss, and recurring disasters lead to psychological distress, depression, and trauma in affected populations (Chhetri, et al., 2024a; Ruby et al., 2025)	Disaster-related trauma, stress prevalence, and psychosocial support availability	Psychosocial & Emotional Stress; Migration & Displacement Pressure
4.	Time Use	Work	Climate disasters disrupt normal work routines, increase recovery time, and require additional labour for resource collection such as water and firewood (Kjellstrom et al., 2017; Nilsson & Kjellstrom, 2010).	Disaster-recovery time, resource-collection duration, and work disruption frequency	Disaster Hazard Exposure; Livelihood & Income Instability
		Sleep	Heatwaves, displacement, and post-disaster stress reduce sleep quality and duration, affecting rest and recovery (Rifkin et al., 2018; Gaston et al., 2023).	Night-time temperature variation, disaster displacement, and stress-induced sleep disruption	Temperature Stress; Psychosocial & Emotional Stress
5.	Education	Literacy	Floods, landslides, and infrastructure damage from extreme weather disrupt access to schools, especially in rural areas, limiting opportunities for literacy development (Katel et al., 2025; Thapa, 2024).	School-closure frequency, infrastructure damage, and education-interruption days	Infrastructure Disruption; Disaster Hazard Exposure
		Schooling	Climate-related disasters cause displacement and reduced school attendance due to damaged facilities and unsafe travel routes (Katel et al., 2025; Thapa, 2024).	Disaster-related absenteeism, education-access stability, and infrastructure recovery time	Infrastructure Disruption; Disaster Hazard Exposure
		Knowledge	Loss of local ecological knowledge from ecosystem degradation weakens communities’ adaptive capacity and climate awareness (Katel et al., 2025; Thapa, 2024).	Traditional-knowledge continuity, climate-literacy level, adaptive-learning inclusion	Ecosystem & Biodiversity Stress; Health & Disease Risk
		Values	Environmental degradation and exposure to climate risks reshape ecological ethics and pro-environmental attitudes among youth (Thapa, 2024; Delma et al., 2024).	Climate-awareness level, ecological-value perception, sustainability-attitude index	Ecosystem & Biodiversity Stress; Psychosocial & Emotional Stress
6.	Cultural diversity & resilience	*Zorig chusum* (local arts and crafts) skills	Scarcity of natural raw materials such as wood, pigments, and bamboo caused by droughts, forest fires, and resource depletion affects the continuity of traditional craftsmanship (Granfelt, 2014; Honwad et al., 2020)	Raw-material availability, forest-resource stress, production loss rate	Ecosystem & Biodiversity Stress; Hydro-climatic Variability
		Cultural participation	Extreme weather events and disasters disrupt religious festivals, community gatherings, and cultural events, reducing participation and continuity of cultural expressions (Garcia, 2019; Granfelt, 2014).	Festival disruption frequency, event-cancellation rate, recovery duration	Disaster Hazard Exposure; Infrastructure Disruption
		Speak native language	Climate-induced migration from rural to urban areas reduces intergenerational language transmission and weakens linguistic diversity (Garcia, 2019; Granfelt, 2014).	Migration-induced language shift, community-retention rate,	Migration & Displacement Pressure
		*Driglam Namzha* (code of etiquettes)	Damage to monasteries and heritage sites from floods and landslides threatens the preservation of etiquette-based practices and traditional learning spaces (Garcia, 2019; Allison, 2004).	Heritage-site vulnerability, restoration frequency	Infrastructure Disruption; Disaster Hazard Exposure
7.	Good governance	Political participation	Climate disasters and displacement reduce citizens’ ability to engage in political processes and community decision-making, particularly in remote and hazard-prone regions (Bisht, 2013; Moore et al., 2024)	Disaster-displacement frequency, participation disruption days	Disaster Hazard Exposure; Migration & Displacement Pressure
		Services	Floods, landslides, and extreme events interrupt delivery of essential public services such as health, education, and utilities, especially in rural communities (Katel et al., 2025; Bisht, 2013)	Service-interruption frequency, infrastructure recovery time, and public-service resilience	Infrastructure Disruption; Disaster Hazard Exposure
		Governance performance	Institutional credibility and trust are influenced by the government’s ability to respond effectively to climate impacts and disasters (Penjor et al., 2022; Katel et al., 2025)	Disaster-response efficiency, adaptation-policy implementation, and institutional-resilience rating	Disaster Hazard Exposure; Livelihood & Income Instability
		Fundamental rights	Relocation, food insecurity, and resource scarcity during disasters threaten equitable access to rights, justice, and security (Katel et al., 2025; Bisht, 2013).	Displacement rate, resource-access inequality, and rights-protection under climate stress	Migration & Displacement Pressure; Livelihood & Income Instability
8.	Community vitality	Donation (time and money)	Recurrent climate disasters increase the need for community donations and volunteerism but also strain household resources, reducing capacity for charitable giving (Yangzom & Wouters, 2024; Phanchung et al., 2022)	Disaster-frequency, volunteer-response rate, and donation-capacity	Disaster Hazard Exposure; Livelihood & Income Instability
		Safety	Extreme weather, landslides, and floods heighten perceived and real threats to life, property, and public spaces, reducing feelings of personal safety (Yangka et al, 2018; Penjor et al., 2022)	Disaster-risk exposure, injury and fatality rate, and infrastructure-vulnerability	Disaster Hazard Exposure; Infrastructure Disruption
		Community relationship	Floods, landslides, and agricultural losses disrupt communal events and traditional cooperation systems, weakening trust and mutual support networks (Gyeltshen et al., 2025; Penjor et al., 2022)	Community-cooperation rate, collective-action participation, and recovery-coordination	Disaster Hazard Exposure; Livelihood & Income Instability
		Family	Climate-induced displacement, migration, and economic stress divide families and reduce intergenerational care and emotional support (Katel et al., 2025; Gyeltshen et al., 2025).	Migration-related separation, livelihood-stress index, and family-support stability	Migration & Displacement Pressure; Livelihood & Income Instability
9.	Living standard	Household per capita income	Climate-related crop loss, livestock mortality, and water scarcity reduce farm income and employment opportunities, particularly in rural and subsistence sectors (Katel et al., 2025; Chhogyel et al., 2020).	Income-loss frequency, agricultural-yield fluctuation, and livelihood diversification	Livelihood & Income Instability; Hydro-climatic Variability
		Assets	Floods, landslides, and windstorms destroy physical assets such as houses, livestock, and productive land, lowering household security and recovery capacity (Chhogyel et al., 2020; Khandu et al., 2022).	Asset-damage index, insurance-coverage level, and asset-recovery time	Disaster Hazard Exposure; Infrastructure Disruption
		Housing	Intense rainfall, heatwaves, and storms damage housing infrastructure, water systems, and sanitation, particularly among low-income families (Chhogyel et al., 2020; Khandu et al., 2022).	Housing-resilience score, storm-damage frequency	Infrastructure Disruption; Temperature Stress; Disaster Hazard Exposure

**Table 3:** The 10 harmonised climate-wellbeing stressors and the grouped climate stressors

	Harmonised climate-wellbeing stressors	Constituents of the harmonised climate-wellbeing stressors comprising thematically grouped climate-related stressors (from column 5, Table 1)
1.	Temperature Stress	Temperature trend; heatwaves; night-time temperature variation; extreme-temperature frequency
2.	Hydro-climatic Variability	Rainfall variability; precipitation intensity; drought; drought duration; water scarcity; water-availability index; fluctuating river flows
3.	Disaster Hazard Exposure	Floods; flood recurrence; landslides; storms; forest fires; forest-fire incidence; GLOF risk; disaster frequency; storm-damage frequency; injury/fatality rate
4.	Glacial & Cryosphere Change	Glacial melt rate; glacial retreat; snowline shift; glacial-lake expansion
5.	Ecosystem & Biodiversity Stress	Habitat degradation; species-range shifts; forest decline; soil-erosion index; river-sediment load; loss of ecosystem services; raw-material availability; forest-resource stress
6.	Livelihood & Income Instability	Crop loss; agricultural yield fluctuation; livestock mortality; income-loss frequency; livelihood security; food price shocks; market disruption; livelihood-stress index
7.	Infrastructure Disruption	Damage to homes, schools, bridges, health facilities; infrastructure damage; service interruption frequency; infrastructure recovery time; damage to heritage sites
8.	Migration & Displacement Pressure	Rural–urban migration; migration pressure; climate-induced relocation; migration-related separation; family fragmentation; community fragmentation
9.	Health & Disease Risk	Heat illness; heatwave exposure days; vector-borne diseases; vector-borne disease incidence; waterborne diseases; pollution exposure; air-quality index
10.	Psychosocial & Emotional Stress	Anxiety, trauma, disaster-related stress; post-disaster emotional recovery; fear; grief from loss; spiritual distress; stress prevalence; coping capacity

To extend the analysis from measurement to policy application, a similar document analysis process was applied to the 23 determinants of the GNH Policy Screening Tool (Penjore, 2008). Using the same search strategy, selection criteria, and analytical steps, literature was reviewed to establish the relationship between each determinant and climate change. Climate stressors defining this relationship were recorded and analysed in NVivo (Table 4). To ensure consistency and avoid duplication, the stressors identified at the determinant level were aligned with the previously developed harmonised climate–wellbeing stressors ensuring a unified analytical framework across both components. Integrating these harmonised climate-wellbeing stressors into the determinants transforms the GNH Policy Screening Tool into a climate-GNH policy screening mechanism. Together, these analyses constitute the empirical foundation for identifying key entry points for integrating climate change into Bhutan’s GNH framework.

**Table 4:** Relationship between 23 determinants of the GNH policy screening tool and climate change, and harmonised climate-wellbeing stressors that could be integrated into each determinant

	Determinant	Relationship with Climate Change	Climate stressors defining the relationship between the determinant and climate change	Harmonised climate-wellbeing stressors to be integrated into the determinant
1.	Pollution	Air pollution and greenhouse-gas emissions contribute to climate change and affect public health (Chhogyel & Kumar, 2018)	Greenhouse-gas emission rate, air-quality index, waste and effluent levels, pollution-exposure days.	Health & Disease Risk; Temperature Stress; Ecosystem & Biodiversity Stress
2.	Biodiversity	Climate change drives habitat loss and species decline, weakening ecosystem services (Banerjee & Bandopadhyay, 2016)	Habitat-fragmentation rate, species-migration trend, ecosystem-restoration progress, forest-cover change	Ecosystem & Biodiversity Stress; Hydro-climatic Variability
3.	Nature	Deforestation, soil degradation, and water scarcity are intensified by climatic shifts (Banerjee & Bandopadhyay, 2016; Yangka et al., 2018)	Forest-cover loss, soil-erosion index, watershed-restoration progress	Ecosystem & Biodiversity Stress; Hydro-climatic Variability
4.	Health	Heat stress, vector-borne diseases, and malnutrition rise with temperature and rainfall changes (Chhetri, et al., 2024a; Ruby et al., 2025)	Temperature anomaly, vector-borne disease incidence, malnutrition prevalence, heatwave exposure days	Health & Disease Risk; Temperature Stress
5.	Security	Floods, droughts, and landslides threaten human safety, food, and water security (Lama et al., 2024; Khandu et al., 2022)	Disaster-frequency, food-security index, water-availability variability	Disaster Hazard Exposure; Hydro-climatic Variability; Livelihood & Income Instability
6.	Productivity	Extreme weather disrupts agriculture, energy supply, and labour capacity (Sharma et al., 2020; Chhogyel et al., 2020)	Agricultural-yield fluctuation, renewable-energy performance, work-disruption frequency	Livelihood & Income Instability; Hydro-climatic Variability; Disaster Hazard Exposure
7.	Equity	Climate impacts are unevenly distributed, widening social and gender inequalities (Yangka et al., 2018)	Gender-differentiated vulnerability, income-loss disparity, access-to-adaptation resources	Livelihood & Income Instability; Migration & Displacement Pressure
8.	Participation	Participatory governance enhances adaptive decision-making and social resilience (Bisht, 2013; Moore et al., 2024)	Stakeholder-engagement rate, local-climate-planning participation, policy-coherence	Governance-related Stress (via Infrastructure Disruption + Disaster Hazard Exposure); Psychosocial & Emotional Stress
9.	Family	Climate migration and economic stress weaken family and community cohesion (Katel et al., 2025; Gyeltshen et al., 2025)	Migration-induced separation rate, livelihood-stress index, family-support stability	Migration & Displacement Pressure; Livelihood & Income Instability; Psychosocial & Emotional Stress
10.	Recreation	Heat and pollution reduce outdoor safety and quality of recreational spaces (Katel et al., 2025; Thapa, 2024)	Urban-heat index, air-pollution exposure, green-space availability	Temperature Stress; Health & Disease Risk; Ecosystem & Biodiversity Stress
11.	Values	Environmental change influences ethical views and sustainability attitudes (Delma et al., 2024).	Climate-awareness level, environmental-ethics perception, sustainability-attitude.	Psychosocial & Emotional Stress; Ecosystem & Biodiversity Stress
12.	Material	Disasters cause loss of assets, housing, and livelihood resources (Katel et al., 2025)	Asset-damage index, housing-resilience score, livelihood-recovery time	Infrastructure Disruption; Disaster Hazard Exposure; Livelihood & Income Instability
13.	Stress	Uncertainty and disaster trauma increase psychological distress (Chhetri, et al., 2024a; Ruby et al., 2025)	Disaster-induced stress prevalence, trauma-recovery duration, access to psychosocial-support services	Psychosocial & Emotional Stress; Disaster Hazard Exposure
14.	Learning	Climate literacy improves preparedness and adaptive capacity (Katel et al., 2025; Thapa, 2024)	Climate-change curriculum coverage, adaptive-learning participation, public-awareness	Ecosystem & Biodiversity Stress; Health & Disease Risk; Hydro-climatic Variability
15.	Information	Access to hazard and early-warning information strengthens community safety (Katel et al., 2025; Thapa, 2024)	Early-warning dissemination rate, communication reach, hazard-information reliability	Disaster Hazard Exposure; Infrastructure Disruption
16.	Anti-corruption	Transparent climate-finance governance builds trust in adaptation programs (Dorji et al., 2025)	Climate-finance transparency, audit frequency, adaptation-fund accountability	Governance-related Stress (subset of Infrastructure Disruption; Disaster Hazard Exposure)
17.	Rights	Displacement and resource scarcity raise risks to property and social rights (Yangka et al., 2018)	Relocation-safeguard effectiveness, property-loss compensation rate, rights-protection mechanisms	Migration & Displacement Pressure; Livelihood & Income Instability
18.	Judiciary Access	Access to justice ensures redress for environmental and climate harms (Turner & Ahuja, 2021)	Grievance-redress frequency, environmental-case resolution time, accessibility of climate-litigation processes	Governance-related Stress (via Infrastructure Disruption)
19.	Judiciary Effectiveness	Enforcement of climate and environmental laws determines compliance (Turner & Ahuja, 2021)	Environmental-law enforcement rate, case-resolution efficiency, climate governance institutional-capacity	Governance-related Stress; Infrastructure Disruption
20.	Culture	Climate hazards threaten heritage sites and cultural continuity (Granfelt, 2014; Honwad et al., 2020)	Heritage-site vulnerability, disaster-damage frequency, restoration-success rate	Infrastructure Disruption; Disaster Hazard Exposure; Ecosystem & Biodiversity Stress
21.	Spirituality	Loss of sacred landscapes affects spiritual wellbeing and environmental ethics (Yangzom & Wouters, 2024; Phanchung et al., 2022)	Sacred-site vulnerability, ritual-disruption frequency, eco-spirituality participation	Psychosocial & Emotional Stress; Infrastructure Disruption; Ecosystem & Biodiversity Stress
22.	Discrimination	Vulnerable groups face disproportionate climate risks and recovery barriers (Johnson, 2023)	Gender- and disability-sensitive adaptation metrics, recovery-time disparities, and inclusion indices	Equity-related: Migration & Displacement Pressure; Livelihood & Income Instability; Health & Disease Risk
23.	Support	Social solidarity and mutual aid enhance community adaptation (Yangzom & Wouters, 2024; Phanchung et al., 2022)	Volunteer-response rate, community-network strength, and social-safety-net coverage	Community Stress: Psychosocial & Emotional Stress; Livelihood & Income Instability; Disaster Hazard Exposure

### Methods in developing global application of wellbeing-centred CRD

In choosing CRD as the mechanism for global application of climate change and wellbeing, a range of existing frameworks with the potential to integrate climate change and wellbeing were considered including sustainable development, resilience thinking, adaptive governance, the wellbeing economy, and Doughnut Economics. However, our choice of CRD was influenced mainly by the IPCC’s Sixth Assessment Report (Schipper et al., 2022; IPCC, 2023), which offers it as an ideal framework towards ecological and human wellbeing (Schipper et al., 2022) and thus provides the conceptual and operational basis for translating Bhutan’s experience into a globally applicable approach.

To operationalise this translation, the empirical findings from Bhutan and the interview data were re-analysed and coded to identify conceptual resonances between GNH and CRD, assess transferability, and identify themes for global application (Braun & Clarke, 2006). For global application, themes representing GNH’s implementation challenges, governance processes, institutional structures, and wellbeing outcomes (Dorji et al., 2025) were systematically compared to CRD’s enabling conditions outlined in the IPCC (Schipper et al., 2022) and global academic literature (Sánchez & Fernández, 2024; Doherty & Clayton, 2011; McPherson & Clarke, 2024; McKenna, 2017; Juhola et al., 2022; Calvet et al., 2022; Adger et al., 2022). The harmonised climate-wellbeing stressors derived earlier provided the substantive content through which climate–wellbeing linkages were interpreted when comparing GNH with CRD. This comparative analysis identified conceptual overlaps, convergent patterns, and gaps between GNH and CRD and it resulted in the emergence of six recurring and mutually reinforcing themes as outlined in the results and discussion section. These themes formed the principal integrative mechanism linking GNH and CRD for global application. Finally, a transferability assessment (Wenger & Olden, 2012) was adopted to evaluate the extent to which insights from Bhutan’s GNH framework can be applied to other contexts, based on criteria such as conceptual resonance, institutional compatibility, and operational feasibility as outlined in the section titled 'limitations and challenges of transferability'.

## Results and discussion

This section presents the findings organised around the two research questions, with interpretation integrated to highlight their analytical and policy relevance.

### Exploring opportunities to integrate climate change into the GNH framework for Bhutan

Two entry points to integrate climate change into the GNH framework were identified; firstly, through the 9 domains and 33 indicators of GNH that guide national policy, and secondly, through the GNH policy screening tools that assess all policies in Bhutan.

#### Integrating climate change into the 9 domains and 33 indicators of GNH

Most (*n* = 35, 85%) of the policy influencers interviewed expressed a clear view that the climate change is conceptually embedded within the GNH philosophy through the pillar of environment conservation, and they did not see climate change as a divorced idea from GNH. However, most agreed that it is time for Bhutan to explicitly and more systematically integrate climate change into the GNH framework. A former prime minister and a global authority on GNH described the GNH index as a dynamic and flexible construct, remarking that:

“The time has come for GNH to address climate change more directly and explicitly. Although the idea of climate change has always been infused into GNH, it was not a central concern. But it should be.”

Interviewees consistently identified the national GNH survey as the most direct and practical entry point for such integration. The survey constructs the GNH Index that guides national policy and planning in Bhutan (Ura et al., 2012). It is organised into 9 domains and 33 indicators representing multidimensional wellbeing (Thinley & Hartz, 2019). Participants explained that if these indicators incorporated climate-related stressors, Bhutan could operationalise climate-responsive wellbeing without altering GNH’s philosophical intent. Building on these interview insights, this study identifies how climate change can be integrated into the GNH measurement framework by linking climate-wellbeing stressors to the existing indicators.

**Fig. 2:**
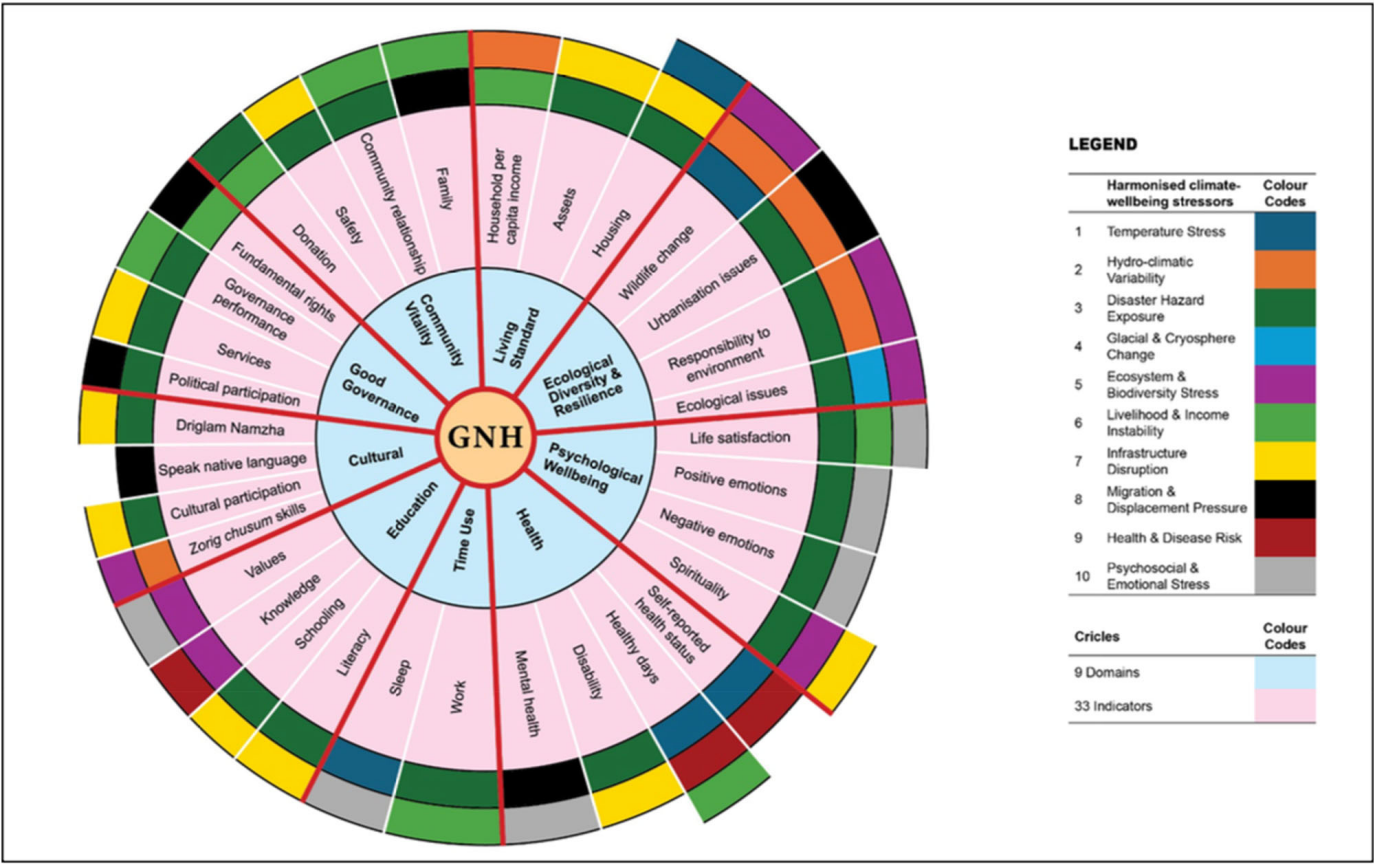
Integration of climate-wellbeing stressors into the 9 domains and 33 GNH indicators: The figure presents a layered structure of the Gross National Happiness (GNH) framework. The innermost circle represents the GNH core, followed by concentric rings displaying the 9 domains and their associated 33 indicators. Surrounding these, are up to three coloured outer rings, which collectively represent the harmonised climate–wellbeing stressors identified in this study. These outer rings do not represent hierarchical levels or distinct categories; rather, they are used for visual clarity to display multiple stressors associated with each indicator. The placement of stressors within these rings does not follow any specific order or organisational logic. Instead, the stressors are distributed across the rings simply to illustrate their relevance to each of the 33 indicators, with each indicator linked to between one and three relevant stressors. The figure therefore demonstrates how climate–wellbeing stressors can be systematically mapped onto the GNH framework, enabling the integration of climate change considerations into all dimensions of wellbeing. (Figure source: developed by authors)

Table 2 presents the outcome of this analysis. Columns 2 and 3 lists the 9 GNH domains and 33 indicators. The fourth column (“relationship with climate change”) summarises the findings on how each indicator is influenced by climate change, while Column 5 lists the climate related stressors influencing this relationship. An examination of these stressors reveals patterns of similarity, overlap, and interconnection across indicators, indicating that climate change affects multiple dimensions of wellbeing in a systemic manner. These overlapping stressors are synthesised into higher-level harmonised climate–wellbeing stressors (Column 6), which provide a structured basis for integration. For example, the harmonised stressor ‘temperature stress’ (row 2 in Table 3) combines temperature trend (from the indicator ‘wildlife change’), heatwaves (self-reported health status), night-time temperature variation (sleep), and extreme-temperature frequency (healthy days). This consolidation highlights how diverse climate impacts converge into common stressors affecting wellbeing. This process reduced the 93 climate stressors identified earlier into the 10 harmonised climate-wellbeing stressors in Table 3. Finally, for each indicator, the relevant climate-wellbeing stressors were mapped resulting in each indicator having 1-3 harmonised stressors for integration (column 6 in Table 2, and Fig. 2).

In this way, all 33 indicators can integrate the harmonised climate-wellbeing stressors that influence wellbeing (Table 2, Fig. 2). The intention here is not merely to list correlations between indicators and climate-wellbeing stressor but to operationalise the process of integration by specifying how it can be embedded within the GNH framework. For example, in the ecological diversity domain, the wildlife change indicator could integrate temperature stress, hydro-climatic variability, and ecosystem and biodiversity. In the psychological wellbeing domain, the life satisfaction indicator could integrate livelihood and income instability; disaster hazard exposure; and psychosocial and emotional stress. Likewise, all the 33 indicators can embed the 10 harmonised climate-wellbeing stressors (Table 2, Fig. 2).

Empirically, our approach demonstrates that climate change interacts with all dimensions of wellbeing, extending beyond the ecological domain. Analytically, it provides a structured evidence base for enhancing the GNH survey instruments into a climate-responsive wellbeing index. These insights highlight that GNH offers an existing institutional scaffold through which climate and wellbeing can be jointly monitored and evaluated, enabling Bhutan to align national happiness metrics with adaptation and resilience objectives as advocated by Thinley & Hartz-Karp (2019) and Ura et al. (2012). Overall, this section demonstrates that climate change can be systematically embedded within each of the 33 indicators—and by extension across all nine domains—to transform the GNH Index into a climate-responsive wellbeing framework. While the current study limits itself to conceptual identification rather than operational execution, it establishes a strong analytical foundation for future research to test and refine these proposed indicators. The following section extends this analysis by examining the second point of integration—the incorporation of climate variables into the GNH Policy Screening Tool—thus moving from measurement to policy application.

#### Integrating climate change into the GNH Policy Screening Tool

Many interviewees pointed out that the next entry point for integrating climate change into the GNH framework is through the GNH Policy Screening Tool. Whereas the first approach embeds climate-wellbeing stressors within the measurement system of the GNH Index, this second focuses on embedding climate sensitivity into Bhutan’s policy-evaluation and decision-making process. Together, they represent complementary mechanisms: one measures climate–wellbeing outcomes, and the other governs policy coherence and implementation.

The GNH Policy Screening Guidelines (Penjore, 2008) stipulate that all new proposed national policies must undergo evaluation before adoption (Ura et al., 2012). A multistakeholder team of experts applies a scoring system, and proposals must reach a minimum threshold score for approval (Thinley & Hartz-Karp, 2019). The tool currently uses 23 determinants that capture cross-sectoral wellbeing dimensions derived from the 9 GNH domains (Penjore, 2008). Although determinants such as pollution, biodiversity, and nature partially reflect environmental concerns, they do not fully account for the wide-ranging impacts of climate change. This limitation provides a clear opportunity to strengthen the tool’s ability to assess climate–wellbeing linkages in policy appraisal.

To operationalise this enhancement, a qualitative document analysis was undertaken to identify climate-wellbeing stressors thematically relevant to each of the 23 determinants. Peer-reviewed academic literature was reviewed to determine how climate change influences each determinant and to identify climate-wellbeing stressors that could be integrated into the existing screening framework. This process mirrored the approach used in exploring integration of climate change into the GNH measurement, where climate-wellbeing stressors were thematically aligned to the 33 GNH indicators, ensuring methodological consistency in both cases. As in the case of integration with the 33 GNH indicators, the initially broad list of climate stressors was consolidated into the same ten harmonised climate-wellbeing stressors to ensure conceptual coherence across the two integration approaches. Using the same harmonised stressor categories for both indicators and determinants enhances analytical clarity and allows the screening tool to evaluate policies against climate impacts in a consistent and non-duplicative manner.

The results are presented in Table 4, which maps the relationship between each determinant and climate change, together with the specific climate-wellbeing stressors to be integrated into each determinant. For instance, the determinant “pollution” can include stressors such as health and disease risk; temperature stress; and ecosystem and biodiversity stress; “biodiversity” and “nature” can integrate ecosystem and biodiversity stress; and hydro-climatic variability. By aligning these with the harmonised stressors, the screening tool can systematically assess how proposed policies influence key climate–wellbeing pressures—such as exposure to hazards, infrastructure disruption, ecosystem degradation, health risks, and psychosocial stress. These additions would enhance the screening tool to assess not only the wellbeing implications of proposed policies but also their climate impacts and contributions to national resilience.

The relationship between the 23 determinants discussed here and the 33 indicators presented earlier is complementary rather than duplicative. The indicators measure population-level wellbeing outcomes, whereas the determinants serve as policy-evaluation criteria derived from those same domains. Aligning the determinants with the climate-wellbeing stressors, therefore, complements the indicator-level integration by ensuring that policies are evaluated for climate–wellbeing coherence, while survey indicators track the resulting impacts. Together, they provide a comprehensive system through which Bhutan can integrate, implement, and monitor climate-responsive wellbeing.

Embedding climate-wellbeing stressors into the Screening Tool would also align Bhutan’s policy architecture with its international obligations. It would directly connect GNH policy evaluation to the Nationally Determined Contributions (NDCs), the National Adaptation Plan (NAP), and Bhutan’s carbon-neutral pledge (Yangka et al., 2018), while reinforcing the principles of the IPCC’s CRD framework that prioritises equity, justice, and human wellbeing (Schipper et al., 2022). This enhancement would transform the GNH Screening Tool into a dual-purpose mechanism—evaluating both wellbeing and climate outcomes—and position Bhutan as a model for integrating national happiness with global climate accountability.

### Global application of wellbeing-centred CRD

Given that GNH is unique to Bhutan (Ura et al., 2012), it is appropriate to consider other relevant mechanisms that might be utilised by other nations to integrate climate change and wellbeing. Different frameworks were considered including sustainable development (Ruggerio, 2021), resilience thinking (Walsh-Dilley et al., 2016), adaptive governance (Munaretto et al., 2014), the wellbeing economy (Hensher, 2023), and Doughnut Economics (Wahlund & Hansen, 2022), but CRD was chosen given its endorsement by the IPCC (Schipper et al., 2022; IPCC, 2023). The IPCC defines CRD as a multidimensional process combining mitigation, adaptation, and sustainable development in an integrated manner to reduce systemic vulnerabilities while enhancing human and ecological wellbeing within planetary limits (Schipper et al., 2022, p. 2657). Both CRD and GNH treat wellbeing not as an outcome but as the core objective of development, consistent with the view of O’Brien and Selboe (2015) that transformative adaptation requires re-imagining what it means to thrive under climate uncertainty. The concept of CRD marks a shift from reactive climate adaptation to the integration of climate action with social justice and sustainable wellbeing (Schipper et al., 2022, IPCC, 2023). This section positions Bhutan’s integration of climate-wellbeing stressors into both measurement (GNH Index) and policy appraisal (Screening Tool) as complementary mechanisms that translate climate–wellbeing linkages into practice. By linking the climate-wellbeing stressors to CRD’s enabling conditions – governance and leadership, metrics, knowledge, resilience, finance, and multi-level integration – Bhutan provides an adaptable model for embedding wellbeing into global CRD.

#### Operationalising a wellbeing-centred approach to CRD

Operationalising a wellbeing-centred approach to CRD requires translating the intersecting climate–wellbeing content presented in Tables 2 and 4 into institutional, policy, and implementation processes. The harmonised climate-wellbeing stressors identified earlier represent the core substantive material through which climate change affects human wellbeing. These stressors outline what must be integrated into CRD, while the six pathways described in this section outline how CRD can be constructed to embed that content. The pathways are sequenced deliberately, beginning with governance and leadership, followed by metrics, knowledge, resilience, finance, and multi-level integration, reflecting a logical progression from institutional foundations to enabling conditions.

First, effective wellbeing-centred CRD requires building institutions for integrative and ethical governance supported by compassionate and values-based leadership. Governance is central to CRD because it determines how risks are prioritised, how adaptation decisions are made, and how justice and wellbeing are upheld (Schipper et al., 2022). Bhutan’s governance framework—including its longstanding emphasis on moral responsibility, collective welfare, and public stewardship—shows how leadership can embed wellbeing in climate-oriented planning (Thinley & Hartz-Karp, 2019). Institutional arrangements that integrate wellbeing into climate policy allow governments to consider the full spectrum of harmonised climate-wellbeing stressors. For example, livelihood and income instability requires governance systems capable of aligning climate adaptation with social protection and economic diversification policies (Sánchez & Fernández, 2024). Similarly, psychosocial and emotional stress calls for institutions that integrate mental-health considerations into disaster recovery and climate communication (Doherty & Clayton, 2011). Leadership that values equity and intergenerational responsibility—core GNH principles—facilitates governance processes that recognise that climate impacts are not merely environmental disruptions but transformations in how people live, relate, and find meaning (McPherson & Clarke, 2024). Such leadership is essential for ensuring that CRD reflects wellbeing-centred values rather than narrow technocratic efficiency.

Second, a practical way of operationalising wellbeing-centred CRD is by embedding wellbeing metrics into climate policy instruments. Metrics make visible the kinds of impacts that must be addressed and provide the foundation for monitoring progress (Ravenscroft et al., 2017). The Bhutan experience shows that the climate-wellbeing stressors presented in Tables 2 and 4 should form the basis of CRD metrics. This ensures that climate-related wellbeing impacts are captured within national climate monitoring systems. Bhutan’s GNH Index provides a model for integrating psychosocial, cultural, ecological, and economic variables into a coherent measurement framework (Ura et al., 2012). Likewise, the GNH Policy Screening Tool shows how policy appraisal can incorporate multidimensional wellbeing considerations (Penjore, 2008). For CRD, metrics derived from the climate-wellbeing stressors offer a way to track the climate–wellbeing interface systematically. They allow countries to assess not only exposure and vulnerability but also emotional security, community vitality, livelihood stability, and ecosystem integrity. Embedding such metrics in CRD also supports evidence-based policymaking aligned with the holistic aspirations articulated in O’Brien and Selboe’s (2015) vision of transformative adaptation.

Third, advancing CRD requires recognising that wellbeing and resilience emerge through knowledge pluralism and the participatory co-production of diverse knowledge systems. Knowledge pluralism refers to the recognition and meaningful integration of multiple ways of knowing—local, experiential, Indigenous, and scientific—to inform more holistic and inclusive understanding and decision-making (McKenna, 2017). As such, it would ensure that CRD is able to respond to climate-wellbeing stressors that are perceived and lived by communities. While scientific knowledge maps temperature trends and precipitation patterns, local and Indigenous Knowledge provides insights about how the physical environmental shifts impact the cultural, emotional and ecological wellbeing (Dorji et al., 2024; Katel et al., 2025). Bhutan offers a compelling example into integrating its spirituality, cultural values and lived experience into environmental governance (Yangka et al., 2018). Globally, empirical evidence shows that local and Indigenous communities have generational knowledge about forest behaviour, ecosystem rhythms, water sources, and social coping strategies (Dorji et al., 2024). Such knowledge is instrumental in addressing migration and displacement pressures or ecosystem and biodiversity stress through culturally informed adaptation strategies (Dorji et al., 2023, 2024). As such, incorporating plural knowledge systems deepens the understanding of human and ecological wellbeing which is crucial for CRD but frequently ignored in technocratic climate policy (Schipper et al., 2022).

Fourth, strengthening CRD requires linking local resilience and global climate frameworks (Juhola et al., 2022). In a wellbeing-centred perspective, resilience refers to the capacity of individuals, communities, ecosystems, and institutions to maintain stability, identity, meaning, and functionality in the face of climate stress (McEvoy et al., 2013). The climate-wellbeing stressors identified in Tables 2 and 4 show that resilience emerges across multiple domains of wellbeing. Local practices in Bhutan, including community forestry, watershed conservation, social cooperation, and cultural rituals, demonstrate how resilience is generated through social cohesion, ecological stewardship, and emotional security (Lama et al., 2024; Khandu et al., 2022). Linking these local expressions of resilience to global frameworks such as the Paris Agreement, the 2030 Agenda, and the Sendai Framework (Schipper et al., 2022) ensures that climate action remains grounded in lived realities while aligning with international commitments. As already discussed, integrating climate-wellbeing stressors into both local adaptation strategies and national reporting systems provides a coherent mechanism for building resilience that is socially meaningful, ecologically grounded, and globally connected.

Fifth, advancing CRD requires transforming climate finance mechanisms to mobilise resources for wellbeing-oriented climate action. Finance is a critical enabling condition because it determines which climate–wellbeing priorities are implemented, scaled, or neglected (Calvet et al., 2022). Conventional climate finance has often privileged carbon efficiency, technological upgrades, or infrastructure protection, while underinvesting in the social, cultural, ecological, and psychological dimensions of climate vulnerability (Bracking & Leffel, 2021). A wellbeing-centred CRD approach requires redirecting financial flows toward livelihood security, mental-health and psychosocial recovery, ecosystem integrity, cultural continuity, and community resilience (Schipper et al., 2022). Integrating the climate-wellbeing stressors from Tables 2 and 4 into financing decisions offers a systematic way to allocate resources to areas of greatest climate-related wellbeing risk. *Bhutan for Life* initiative illustrates how financing aligned with wellbeing priorities can simultaneously safeguard ecological assets and strengthen social and economic wellbeing (Lham et al., 2018). In this way, climate finance becomes the mechanism that enables the substantive climate–wellbeing integration outlined earlier to be realised in practice.

Finally, strengthening CRD requires multi-level approach linking climate–wellbeing integration across local, national, and global systems. Multi-level integration is essential because climate impacts and wellbeing outcomes materialise differently across scales, and coherent governance ensures that these differences are recognised in planning and implementation (Adger et al., 2022). The climate-wellbeing stressors identified in Tables 2 and 4 must be embedded consistently across community action, national policy instruments, and global reporting frameworks so that monitoring systems, financing decisions, and resilience strategies remain aligned. Climate impacts vary by level: community-level psychosocial and emotional stress may contrast sharply with national concerns around livelihood and income instability, while infrastructure disruption may affect urban and rural settings in divergent ways (Thompson et al., 2013; Yangzom & Wouters, 2024). Ecosystem degradation can also cross administrative boundaries, undermining conventional governance lines (Thompson et al., 2013). A multi-level approach reduces trade-offs, enhances co-benefits, and ensures that wellbeing-centred CRD reflects both lived climate realities and national strategic priorities. This strengthens the capacity of NDCs, NAPs, and carbon-neutral commitments to remain grounded in local vulnerabilities while ensuring coherence with global climate frameworks such as the Paris Agreement and the Sendai Framework.

**Fig. 3:**
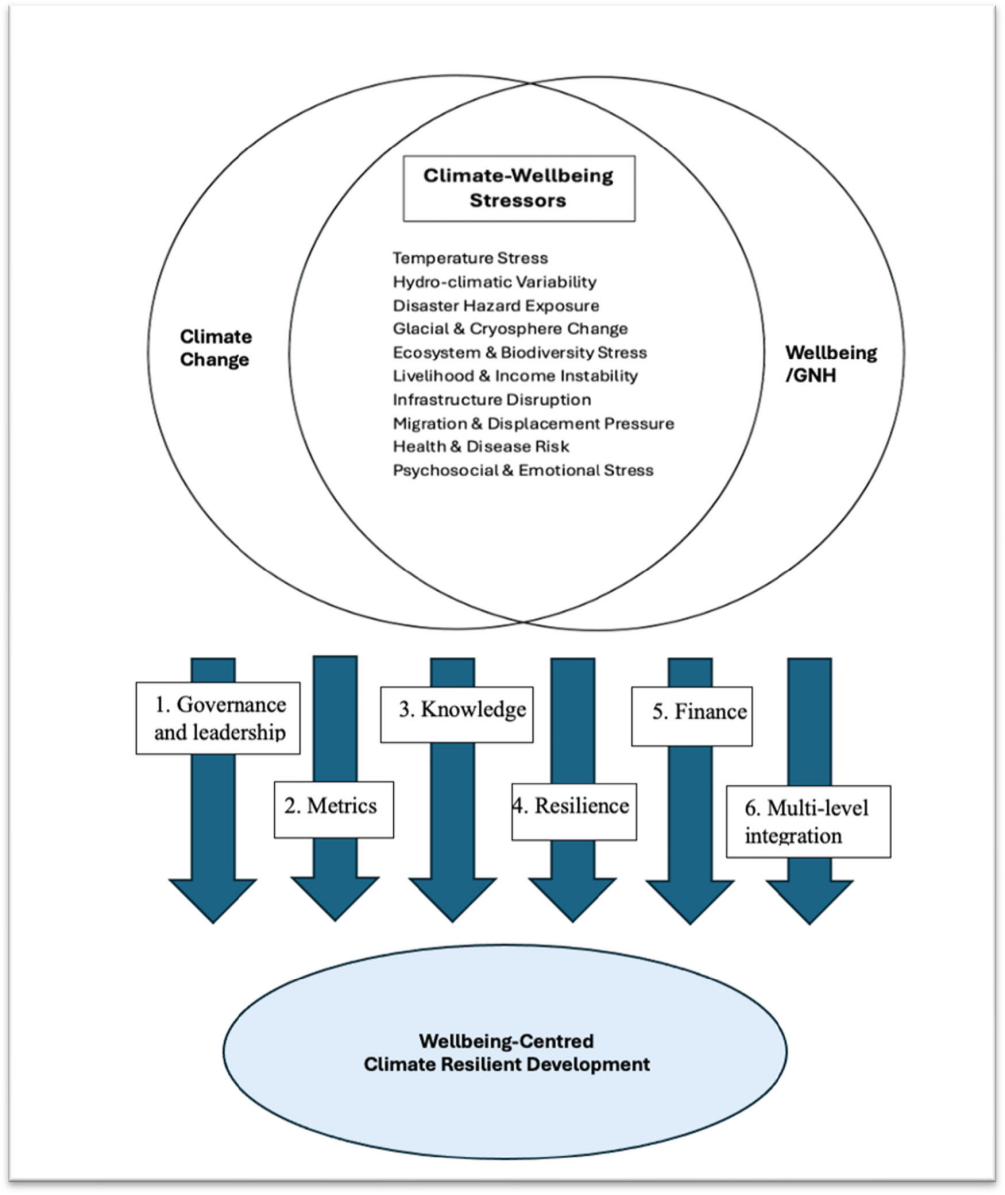
Operational pathways for a wellbeing-centred approach to CRD: The figure illustrates the six pathways identified for operationalising a wellbeing-centred approach to CRD. These pathways represent the institutional, policy, and systemic mechanisms required to translate wellbeing priorities into climate action. Together, they show how Bhutan’s experience can inform a coherent framework for advancing wellbeing-centred climate action in global contexts. (Figure source: developed by authors)

The six pathways (illustrated in Fig. 3) demonstrate how the climate-wellbeing stressors identified earlier can be systematically embedded within the institutional and operational dimensions of CRD for global application. By aligning governance and leadership, metrics, knowledge, resilience, finance, and multi-level integration, this framework shows how wellbeing can become a practical organising principle rather than an aspirational ideal. The Bhutan case illustrates that integrating climate–wellbeing linkages requires both substantive clarity about the nature of climate impacts and coherent mechanisms for acting on them. Operationalised in this way, wellbeing-centred CRD offers an adaptable model that strengthens equity, responsiveness, and long-term resilience, while ensuring that climate action remains grounded in the lived realities of communities and ecosystems.

#### Limitations and challenges of transferability

The application of Bhutan’s GNH experiences into global CRD practice is not without challenges despite its conceptual resonances. Firstly, the moral foundations of GNH are rooted in Buddhist philosophy and governance culture and it may not be compatible with other pluralist or market-driven societies and governance models that are divorced from their ethical foundations. Secondly, the technical, administrative, and financial resources needed to implement wellbeing-centred CRD are significant and still differ between countries (Braunschweiger & Pütz, 2021). Bhutan’s own experience shows that supporting the philosophical intent of operationalising GNH requires strong operational tools (Dorji et al., 2025). Thirdly, resilience indicators frequently ignore intangible elements like trust, meaning, and belonging, making it intrinsically challenging to measure wellbeing without erasing cultural nuances (Schipper et al., 2022). Finally, replacing GDP with wellbeing metrics could invite political resistance and as such, institutionalising wellbeing indicators into national planning, legislation, and budgetary system could help secure the continuity and legitimacy of CRD beyond political objectives (Averchenkova et al., 2017). Therefore, the application of wellbeing-centred CRD globally calls not for replication of Bhutan’s model but for contextual adaptation—embedding wellbeing-centred principles within climate policy frameworks that reflect diverse governance systems, social values, and cultural realities.

## Conclusion

This study examined how climate change can be systematically integrated into Bhutan’s development philosophy of GNH and how this experience can inform a wellbeing-centred CRD for global application. This paper first identified practical entry points for integration within Bhutan—specifically through embedding climate-wellbeing stressors into the 9 GNH domains and 33 indicators that guide national policy, and into the 23 determinants of the GNH Policy Screening Tool used for assessing policies. These two points of integration demonstrate how climate change considerations can be mainstreamed into Bhutan’s wellbeing architecture without altering its philosophical core.

Drawing on Bhutan’s experience, this study shows that wellbeing-centred CRD can be operationalised through six interconnected pathways for global application. Strengthening governance and leadership provides the moral and institutional basis for equitable climate action. Embedding wellbeing metrics into climate policy instruments ensure that climate–wellbeing impacts are systematically recognised and acted upon. Knowledge pluralism ensures culturally meaningful adaptation. Linking local resilience to global frameworks aligns community priorities with international commitments. Wellbeing-oriented finance directs resources to areas of greatest vulnerability. Finally, multi-level integration ensures coherence across scales, grounding CRD in lived realities while maintaining global alignment. Together, these pathways show that CRD can be achieved in its spirit of equity and social justice only when it is meaningfully integrated with wellbeing principles. This study positions GNH as a globally relevant guide for transforming climate action towards human and planetary flourishing and offers an integrative approach for nations pursuing wellbeing objectives to combine it with climate action for holistic development. Its global relevance lies in offering a wellbeing-centred approach to CRD.

## Data Availability

All data generated or analyzed during this study are included in this published article.
